# Draft genome sequence of *Rhodococcus erythropolis* DR10, a potential oil degradation actinobacteria

**DOI:** 10.1128/mra.01153-25

**Published:** 2026-03-10

**Authors:** Puja Gupta, Jyoti Vakhlu, Pradeep Kumar, Sundeep Jaglan

**Affiliations:** 1 School of Biosciences, RIMT University 471390, Punjab, India; 2 School of Biotechnology, University of Jammu 29074, Jammu, India; 3 Department of Microbiology, Kalinga University, Naya Raipur, Chhattisgarh, India; 4 Microbial Biotechnology Division, CSIR-Indian Institute of Integrative Medicine 81690, Jammu and Kashmir, India; University of Southern California, Los Angeles, California, USA

**Keywords:** *Rhodococcus erythropolis*, genome sequencing, oil degradation

## Abstract

We hereby report the draft genome sequence of *Rhodococcus erythropolis* DR10, an actinobacterium isolated from the soil of Ladakh via minimal media agar. The genome 6,395,202 bp exhibited a 62.5% GC content with 5,883 coding sequences, 1 rRNA, and 53 tRNAs.

## ANNOUNCEMENT

*Rhodococcus erythropolis* DR10 is a gram-positive actinobacterium isolated from the composite soil sample of Drass (Ladakh), located at 34.428152°N, 75.75118°E, using minimal media agar. The isolation and molecular identification of strain DR10 followed the methods described in the previous study (1). Here, we report the draft genome sequence of *R. erythropolis* DR10 to highlight its potential biotechnological relevance.

The bacterial isolate DR10 was grown on R2A agar, and a single colony was picked, followed by genomic DNA extraction using HiPurA Kit (cat. no. MB505). The latter was amplified using 16S rDNA universal primers (Bac8f and Univ529r) (2), sequenced, and searched for sequence similarity against the nucleotide database using BLASTN. The 16S rDNA sequence of strain DR10 (GenBank accession number JX978890.1) showed 99.8% similarity with *Rhodococcus erythropolis* strain XP.

Whole-genome paired-end sequencing (2 × 150 bp) was performed on the Illumina (MiSeq platform), producing 5,785,102 paired-end reads. Genomic DNA was further fragmented, end-repaired, and A-tailed, followed by ligation with Illumina paired-end adapters using NEBNext Ultra TM II FS DNA Library Prep Kit for Illumina (New England BioLabs, USA). PCR enrichment and quality control (Qubit and Agilent TapeStation) were performed. The resulting PE library was loaded onto an Illumina sequencing platform for cluster generation and subsequent sequencing of both ends.

Quality control and trimming were performed using Fastp v0.201 (3), assembled with Shovill v1.0.4 (4), producing a single contig of 6,395,202 bp. Genome annotation was performed by Prokaryotic Genome Annotation Pipeline (5). The genome of strain Dr10 was assembled as one circular chromosome without a plasmid, with a total length of 6,395,202 bp, accomplishing 170× coverage of the genome. The genome comprised 5,883 protein-coding genes, 53 tRNA genes, 1 rRNA gene, and an average GC content of 62.5%. Circularization of the contigs was performed by overlapping terminal sequences. The circularization of the chromosome was confirmed by determining the same sequences at both ends of the contig using BLASTN. Furthermore, the annotation of the genome indicated the availability of genes that are associated with hydrocarbon degradation, which could prove its applicability in oil degradation.

Default parameters were used for all software unless otherwise specified.

## Data Availability

The whole genome of *R. erythropolis* Dr10 has been deposited to NCBI under submission ID JBREAP010000001, Project ID PRJNA1299411, Biosample ID SAMN50294882, and Sequence Read Archive no. SRR34790003.
